# Sociodemographic, environmental and labor conditions related to the presence of conjunctivitis and skin irritation in a group of informal street vendors in downtown Medellin, 2015-2019

**DOI:** 10.47626/1679-4435-2021-525

**Published:** 2021-04-30

**Authors:** María Osley Garzón Duque, Sebastián García, Daniel Tamayo, Doris Cardona Arango, Ángela María Segura Cardona, Fabio León Rodríguez Ospina, Catalina Betancur Vasquez, Diego Alejandro Marsiglia

**Affiliations:** 1 Facultad de Medicina, Universidad CES, Medellín, Antioquia, Colombia; 2 Facultad de Medicina, Escuela de Graduados, Universidad CES, Medellín, Antioquia, Colombia; 3 Escuela de Graduados, Universidad CES, Medellín, Antioquia, Colombia; 4 Facultad Nacional de Salud Pública, Universidad de Antioquia, Medellín, Antioquia, Colombia

**Keywords:** conjunctivitis, dermatitis, occupational health, environmental pollution, informal work

## Abstract

**Introduction:**

Occupational diseases are those that may have a causal relationship with occupational activity or environment. However, this definition does not specify how this disease would be identified and acknowledged for workers with subsistence jobs.

**Objectives:**

To determine sociodemographic, labor and environmental conditions that collaborate to explain the presence of eye and skin irritation among informal vendors in downtown Medellin.

**Methods:**

Descriptive cross-sectional study with analytical intention, primary sources of information, and administration of assisted survey including self-reported eye and skin irritation in 686 workers.

**Results:**

Predominantly male population with mean age of 50 (±11.8) years. A total of 23.5 % of workers had worked as vendors for their entire life; 37.5% had worked as vendors for 11 to 20 years as vendors; and 81.5%worked for more than 8 hours a day. According to 69.8% of the sample, poor air quality affected their work, and 80.0% believed that pollution was generated by vehicle fleet. The polluted areas generated unpleasant odors (50.2%) and air pollution (89.4%). The prevalence of eye and skin irritation was 65.4%. Not having working license and having fair, poor, or very poor job tenure significantly reduced the prevalence of eye and skin irritation. Conversely, exposure to polluted water, working in the professional for more than 30 years, male sex, and age older than 60 years were associated with increased prevalence of irritation.

**Conclusions:**

Eye and skin irritation was mainly explained by non-modifiable sociodemographic and labor conditions and by consumption of polluted water; however, implementing public health actions could reduce workers’ socio-environmental and labor vulnerability.

## INTRODUCTION

Occupational diseases are defined as those resulting from the exposure to risk factors inherent to the occupational activity or environment, according to the article 4 of Law 1562, which came into effect in 2012 in Colombia,^1^ as well as those that may present a causal relationship with these factors. However, this definition does not specify how these diseases would be identified and acknowledged in the informal sector of economy.

In Latin America, occupational diseases and accidents lead to high morbidity and mortality rates in the formal sector.^2^ In Colombia, according to the 2018 report of the Federación de Aseguradores Colombianos,^3^ “the rate of occupational diseases decreased 24.0% in the last eight years, reducing from 130.6/100,000 to 99.6/100,000”.^3^ Nevertheless, this information is not available yet for individuals working in the informal economy, among which morbidity and mortality rates could often result from their precarious labor conditions and exert a great impact on their physical and mental health status.^4^

In July 2019, 47.5% of the Colombian worker population had informal jobs,^5^ meaning that this form of employment is the means of subsistence for a high percentage of people. However, there is still little information on the morbidity conditions related to air pollution among these workers, particularly in the city of Medellín,^6^ whose downtown district^7^ is one of the areas with the greatest amount of informal workers and the highest environmental pollution of the metropolitan area of the Aburra Valley, as shown by the reports of Contraloría General de Medellín and of the Instituto de Hidrología y Meteorología (IDEAM). According to these reports, in 2016 the concentrations of particulate matter exceeded the maximum allowable threshold in 6 of the 9 monitoring stations that complied with the temporal representativeness criterion. The maximum category of the Air Quality Index (AQI) achieved during 2016, corresponding to hazardous levels for the health of sensitive groups, was reported in the monitoring stations Éxito San Antonio and Museo de Antioquía. However, the highest number of stations showed a moderate AQI level, ranging from 56.8% to 74.1%.^8,9^

These locations concentrate a high amount of informal street vendors working in the downtown district, where they are exposed to situations and pollutants (high levels of noise, heat, particulate matter, and gases)^10^ that may be affecting their physical health status, particularly because studies with this type of population have already shown that their working days are long and they have low monthly income.^6,11^

One of the physical diseases that could arise among informal workers in downtown Medellin is eye and skin irritation (ESI), which could be related to environmental pollution. However, current evidence on the theme is not conclusive, because it is little explored within this population. Similarly, there is no conclusive information on how this type of health illnesses could be explained by the sociodemographic, labor, and environmental conditions in which workers develop their work,^12^ which explains the relevance of providing evidence on this matter. Therefore, the present study aimed to determine which sociodemographic, working, and environmental conditions explain the presence of ESI in a group of informal street vendors working in downtown Medellin, in order to acknowledge modifiable and non-modifiable risk factors, which could be approached by actions and public health interventions that contribute to improve workers’ physical health conditions,^13^ and contribute to minimize their conditions of socio-environmental and labor vulnerability.

## METHODS

### DESIGN

The present study is a subproject of the doctoral thesis entitled “Condiciones ambientales, laborales, sociodemográficas, económicas y de percepción de salud que configuran la condición de vulnerabilidad laboral de un grupo de trabajadores informales ‘venteros’ del centro de Medellín, 2015-2019” and was approved by the institutional Ethics Committee of Universidad CES, Minutes 84, dated September 24, 2015.

### POPULATION

Survey with 686 informal street vendors working in downtown Medellin.

### VARIABLES

- Outcomes: prevalence of ESI. The following sociodemographic variables were considered explanatory variables: sex, age, and marital status.
- Labor conditions: type of vendor, type of product, daily working hours, years working in the profession, working license, previous occupation, job tenure, conditions of working tools, order and cleanliness of point of sale and its surroundings, feeling of exaggerated heat or cold, use of chemicals, exposure to chemicals, possible contamination with microbes in the working area, personal protection methods, damaged walls and floor.
- Environmental conditions: time of day when the respondent considers air pollution is greater, time of exposure to the pollutant, air quality affects work, point of sale near polluted area or site; waste accumulation in open spaces, waste accumulation in stationary waste buckets; waste accumulation in containers, sewage source, market place, vehicle fleet or restaurant, the polluted area or site generates: unpleasant odors, visual pollution, air pollution, water pollution, among others.

### PILOT TEST, CONTROL OF ERRORS AND BIASES

A pilot test was conducted prior to administering the instrument, and the principal investigator and her field assistant were standardized for data collection. Selection bias was controlled by using a census sampling technique.

### ANALYSIS

The nature and the measurement level of the variables were considered. A normality test was applied to assess data distribution for quantitative variables, and frequency and percentage were calculated for qualitative variables in univariate analyses. Non-causal associations between explanatory variables and prevalence of ESI were explored using the chi-square test, and prevalence ratio (PR) and its respective 95% confidence interval (95%CI) were used as epidemiological measurements. The types of products were reclassified into: seasonal and perishable products; beverages, appetizers, and desserts; fast food; goods and pots, with the latter being the reference category. Moreover, sociodemographic, labor, and environmental conditions were reclassified for bivariate and multivariate analyses.

- Multivariate analysis with multiple logistic regression, in order to identify the factors that contribute to explain ESI: variables were included in the model from the lowest to the highest p-value, according to the results of bivariate analysis and complying with the Hosmen-Lemenchow criterion (p < 0.25). Tests were applied considering a 95%CI and a significance level of 5.0%. Calculations were made using Epidat 3.1, 4.1 and SPSS® version 21 (licensed to Universidad CES). Table and text layouts were created using Microsoft Excel and Word.

## RESULTS

### SOCIODEMOGRAPHIC AND LABOR CONDITIONS

Workers’ mean age was 50 years (± 11.76), with a greater proportion of men (57.6%) and individuals without a partner (52.4%) (Table 1).

**Table 1:** Proportional distribution of sociodemographic and labor conditions among informal vendors participating in the study, Medellin, Colombia, 2015-2019

Characteristic or condition	n	%
Sociodemographic conditions		
Sex		
Male	395	57.7
Female	291	42.3
Reclassified age (years)		
18 to 29	29	4.2
30 to 44	190	27.7
45 to 59	314	45.8
≥ 60	153	22.3
Marital status		
Without a partner	358	52.4
With a partner	325	47.6
		
Labor conditions		
Daily working hours		
4 to 8	127	18.5
> 8	559	81.5
Length of time working in the profession (years)		
5 to 10	81	11.8
11 to 20	257	37.5
21 to 30	190	27.7
> 30	158	23.0
Previous occupation		
Vendor has been their only occupation	161	23.5
Laborer, farmer, housewife, employee	525	76.5
Working license		
No	266	38.8
Yes	420	61.2
Job tenure		
Fair, poor, very poor	180	26.3
Very good, good	504	73.6
Type of vendor*		
Stationary	122	17.8
Semi-stationary or itinerant	564	82.2
Type of product^†^		
Goods and pots	403	58.8
Other products^‡^	282	41.2
		
Occupational risk factors		
Deteriorated walls and floors		
Yes	244	35.6
No	442	64.4
Working tools in good conditions		
Yes	400	58.3
No	267	40.4
Not applicable	9	1.3
Clean and tidy working area		
Yes	644	93.9
No	42	6.1
Chemicals producing sickness in the workplace		
Yes	138	20.2
No	548	79.8
Possible contamination with microbes		
Yes	397	57.9
No	289	42.1
Use of personal protection measures		
Yes	187	27.3
No	499	72.7
Feeling of exaggerated heat or cold in the workplace		
Yes	587	86.6
No	99	14.4

* Type of vendor: stationary when the vendor remains at the same workplace 24 hours a day; semi-stationary when the workplace can be located in the public space only during vendor's working hours; and itinerant when the goods or the products sold by the vendor are carried on his/her body.

† Type of product: goods and pots; seasonal and perishable products; beverages, appetizers, and desserts; and fast food.

‡ Other products: seasonal and perishable products; fast food; beverages, appetizers, and desserts.

With regard to labor conditions, 23.5 % (161) of participants had only worked as a street vendor during all their lives; most (37.5 %) had been working for 11 to 20 years; and most worked more than 8 hours a day (81.5%). Furthermore, 61.2% (420) had a working license and more than 75.0% considered they had job tenure. Study participants were mostly semi-stationary and itinerant vendors, and the highest proportion of workers (58.8%) sold goods and pots (Table 1).

When it comes to occupational risk factors, 40.4% (277) of participants considered that their working tools were not in good conditions; 35.6% (244) believed that there were damaged walls or floors in their workplace or in its surroundings; 6.1% (42) stated that their working area was not clean and tidy. Furthermore, 72.7% (499) of workers reported not using personal protection measures; 57.9% (397) believe that they could be contaminated with microbes in the workplace or in its surroundings; and 86.6% (587) stated that they were exposed to exaggerated heat or cold in the workplace (Table 1).

### ENVIRONMENTAL CONDITIONS IN THE POINT OF SALE AND ITS SURROUNDINGS

A total of 69.8% (479) believed that poor air quality affected their work, 56.4% (373) reported that morning and evening were the periods of the day with greater air pollution; 36.0% (220) observed waste accumulation in open spaces in the workplace or its surrounding; 34.9% (213) considered to be near wastewater (sewage); 80.0% (489) stated that the polluted area or site (contaminant source) was represented by el vehicle fleet. These pollutant sources mainly generated unpleasant odors (50.2%) and air pollution (89.4%) (Table 2).

**Table 2:** Proportional distribution of environmental conditions and polluting sources in the workplaces of informal street vendor participating in the study, and prevalence of eye and skin irritation, Medellin, Colombia, 2015-2019

Characteristic or condition	n	%
Environmental conditions		
Air quality affects vendor's work		
Yes	479	69.8
No	207	30.1
Period of the day with the greatest pollution		
Noon and afternoon	312	45.5
Morning, evening, the entire day	373	56.4
Time of exposure to the pollutant (hours)		
< 10	168	27.5
6 to 10	396	64.8
1 to 5	47	7.7
Chemicals are used in the workplace surroundings		
Yes	208	30.3
No	478	69.7
		
Polluted area or site (polluting sources) (n = 611)		
Waste accumulation in open spaces		
Yes	220	36.0
No	391	64.0
Waste accumulation in stationary waste buckets		
Yes	64	10.5
No	547	89.5
Waste accumulation in containers provided by EEVV in the public space*		
Yes	11	1.6
No	600	87.5
Source of wastewater (sewage)		
Yes	213	34.9
No	391	65.1
Marketplace		
Yes	23	3.8
No	588	96.2
Vehicle feet		
Yes	489	80.0
No	122	20.0
Restaurants		
Yes	17	2.8
No	594	97.2
		
Type of pollution generated by the contaminated area or site (pollutant source)		
Unpleasant odors		
Yes	307	50.2
No	304	49.8
Visual pollution		
Yes	63	10.3
No	548	89.7
Air pollution		
Yes	546	89.4
No	65	10.6
Water pollution		
Yes	19	3.1
No	592	96.9
Other (smoke, vapors, noise, etc.)		
Yes	18	2.9
No	593	97.1
		
Outcome (n = 372)		
Eye and skin irritation		
Yes	244	65.4
No	128	34.3

* EEVV = Empresas Varias de Medellín: autonomous organization of the City of Medellin responsible for delivering cleaning services, understood as the waste collection (especially solid waste) and supplemental activities of transportation, sweeping, and cleaning of public routes and spaces, treatment, utilization, and final disposal of waste, lawn mowing, and trimming of trees located in public routes and spaces).

### PREVALENCE OF ESI

A total of 54.4% of the workers reported that, in the six months prior to the survey, they had presented with some disease related to air contamination; of these, 65.4% (244) mentioned that they have developed ESI (Table 2).

### SOCIODEMOGRAPHIC AND LABOR CONDITIONS ASSOCIATED WITH ESI

There was a statistically significant association (p < 0.05) of ESI with working license and job tenure. That is, workers with working license had a higher prevalence of ESI than those with no license (PR = 0.81; 95%CI = 0.68-0.95); whereas, for each vendor with good and very good job tenure who had ESI, 0.81 workers with fair, poor, and very poor job tenure who had the same condition (PR = 0.81; 95%CI = 0.68- 0.97) (Table 3).

**Table 3:** Sociodemographic and labor conditions associated with the presence of eye and skin infection in informal street vendors participating in the study, Medellin, Colombia, 2015-2019

Characteristic or factor	Eye and skin irritation		Total	Chi-square test (p-value)	PR (95%CI)
	Yes (n, %)	No (n, %)			
Sociodemographic characteristics					
Sex					
Male	144 (69.20)	64 (30.8)	208	2.77 (0.096)	1.13 (0.97-1.32)
Female	100 (61.00)	64 (39.0)	164		1
Age (years)					
18-29	11 (57.90)	8 (42.10)	19	2.13 (0.144)	1
30-44	62 (60.20)	41 (39.8)	103		1.03 (0.68-1.57)
45-59	114 (68.30)	53 (31.70)	167		1.17 (0.79-1.75)
> 60	57 (68.70)	26 (31.30)	83		1.18 (0.78-1.78)
Marital status					
Without a partner	103 (64.00)	58(36.00)	162	0.32(0.56)	0.95 (0.82-1.12)
With a partner	141 (66.80)	70 (33.20)	211		1
					
Labor conditions and risk factors					
Daily working hours					
4 to 8	40 (58.00)	29 (42.00)	69	2.17 (0.13)	0.86 (0.69-1.06)
> 8	204 (67.30)	99 (32.70)	303		1
Length of time working in the profession (years)					
5 to 10	30 (61.20)	19 (38.80)	49	1.91 (0.16)	1
11 to 20	82 (59.90)	55 (40.10)	137		0.97 (0.75-1.27)
21 to 30	78 (75.00)	26 (25.00)	104		1.22 (0.95-1.57)
> 30	54 (65.90)	28 (34.10)	82		1.07 (0.81-1.41)
Previous occupation					
Only occupation - vendor	51 (58.60)	36 (41.40)	87	2.44 (0.11)	0.86 (0.71-1.05)
Laborer, farmer, housewife, employee	193 (67.70)	92 (32.30)	285		1
Working license					
No	82 (57.30)	61 (42.70)	143	7.00 (0.008)	0.81 (0.68-0.95)
Yes	162 (70.70)	67 (29.30)	229		1
Job tenure					
Fair, poor, very poor	66 (56.90)	50 (43.10)	116	5.64 (0.017)	0.81 (0.68-0.97)
Good, very good	178 (69.50)	78 (30.50)	256		1
Type of vendor					
Stationary	44 (66.70)	22 (33.30)	66	0.04 (0.83)	1.02 (0.84-1.23)
Semi-stationary or itinerant	200 (65.40)	106 (34.60)	306		1
Type of product					
Goods and pots	149 (65.10)	80 (34.90)	229	0.073 (0.78)	0.97 (0.82-1.15)
Other products	95 (66.40)	48 (33.60)	143		1
					
Occupational risk factors					
Damaged walls and floors					
Yes	89 (63.10)	52 (36.90)	141	0.61 (0.43)	0.94 (0.80-1.09)
No	155 (67.10)	76 (32.90)	231		1
Working tools in good conditions					
Yes	152 (67.90)	72 (31.10)	224	0.36 (0.54)	1
No	84 (60.40)	55 (39.60)	139		0.89 (0.75-1.04)
Not applicable	8 (88.90)	1 (1.1)	9		1.3 (1.02-1.67)
Clean and tidy area					
Yes	220 (64.90)	119 (35.10)	339	0.81 (0.36)	0.89 (0.71-1.11)
No	24 (72.70)	9 (27.30)	33		1
Chemicals producing sickness in the workplace					
Yes	23 (62.20)	14 (37.80)	37	0.21 (0.64)	0.94 (0.72-1.12)
No	221 (66.00)	114 (34.00)	335		1
Possible contamination with microbes					
Yes	115 (64.50)	66 (36.50)	181	0.65 (0.41)	0.94 (0.81-1.09)
No	129 (67.50)	62 (32.50)	191		1
Use of personal protection measures in the workplace					
Yes	66 (64.70)	36 (35.30)	102	0.03 (0.84)	0.98 (0.83-1.16)
No	175 (65.80)	91 (34.20)	266		1
Feeling of exaggerated heat and cold in the workplace					
Yes	225 (66.00)	116 (34.00)	341	0.27 (0.59)	1.07 (0.8-1.43)
No	19 (61.30)	12 (38.70)	31		1

95%CI = 95% confidence interval; PR = prevalence ratio. Values in bold: statistically significant association when p < 0.05.

Although no statistically significant association was observed between age and the outcome of interest, there was a higher prevalence of ESI (18%) among workers aged 60 years or older than among those aged from 18 to 29 years (PR = 1.18). There was also a higher prevalence of ESI among those who had worked in the profession from 21 to 30 years (PR = 1.22). Furthermore, the prevalence of ESI was higher in men (PR = 1.13) (Table 3).

### ENVIRONMENTAL CONDITIONS ASSOCIATED WITH ESI

Statistical significant associations (p < 0.05) were observed between waste accumulation in containers provided by Empresas Varias de Medellín (EEVV) in the public space and air pollution, meaning that, for each worker with ESI who were not near waste accumulated in containers provided by EEVV, there were 0.48 workers under the same condition who presented with the disease and were near this type of waste. That is, being near waste accumulated in EEVV containers reduces the prevalence of ESI by 52.0% (PR = 0.48; 95%CI = 0.25-0.95). Moreover, for each worker reporting to have had ESI who considered that the air in the workplace was not polluted, there were 0.74 workers presenting with this condition who believed that the air in the workplace was polluted. Additionally, a higher prevalence of ESI was observed among workers who stated being near polluted water (PR = 1.27) and near other pollutants, such as smoke, near the workplace (PR: 1.16) (Table 4).

**Table 4:** Environmental conditions and pollutant sources associated with presence of eye and skin irritation in informal street vendor participating in the study, Medellin, Colombia, 2015-2019

Characteristic or factor	Eye and skin irritation		Total	Chi-square (p-value)	PR (95%CI)
	Yes (n, %)	No (n, %)			
Environmental conditions					
Air quality affects work					
Yes	190 (64.20)	106 (35.80)	296	1.260 (0.26)	0.90 (0.76-1.06)
No	54 (71.10)	22 (28.90)	76		1
Period of the day with greater pollution					
Noon and afternoon	115 (65.00)	62 (35.00)	217	1.330 (0.24)	1.07(0.94-1.23)
Morning, evening, the entire day	129 (66.20)	66 (33.80)	195		1
Time of exposure to the pollutant					
More than 10 hours	61 (67.00)	30 (33.00)	91	0.010 (0.99)	1
6 to 10 hours	166 (64.60)	91 (35.40)	257		0.96 (0.81-1.14)
1 to 5 hours	17 (70.80)	7 (29.20)	24		1.05 (0.78-1.41)
Use of chemicals near the workplace					
Yes	77 (68.80)	35 (31.30)	112	0.700 (0.40)	1.07 (0.91-1.24)
No	167 (64.20)	93 (35.80)	260		1
					
Polluted area or site (pollutant sources)					
Waste accumulation in open spaces					
Yes	80 (63.50)	46 (36.50)	126	0.090 (0.76)	0.97 (0.82-1.14)
No	140 (61.50)	75 (34.90)	215		1
Waste accumulation in stationary waste bucket					
Yes	24 (64.90)	13 (35.10)	37	0.0022 0.002 (0.96)	1.00 (0.78-1.29)
No	196 (64.50)	108 (35.5)	304		1
Waste accumulation in containers provided by EEVV in the public space					
Yes	6 (66.70)	13 (33.30)	19	8.300 (0.004)	0.48 (0.25-0.95)
No	214 (64.50)	118 (35.50)	332		1
Source of wastewater (sewage)					
Yes	6 (66.70)	3 (33.30)	9	0.010 (0.89)	1.03 (0.64-1.65)
No	214 (64.50)	118 (35.50)	332		1
Marketplace					
Yes	82 (62.60)	49 (37.40)	131	0.340 (0.55)	0.95 (0.80-1.12)
No	138 (65.70)	72 (34.30)	210		1
Vehicle fleet					
Yes	182 (64.50)	100 (35.50)	282	0.0004 0,000 (0.98)	1.00 (0.81-1.23)
No	38 (64.40)	21 (35.60)	59		1
Restaurants					
Yes	10 (71.40)	4 (28.60)	14	0.300 (0.58)	1.11 (0.79-1.56)
No	210 (64.20)	117 (35.80)	327		1
					
Type of pollution generating polluted area or site (polluting source)					
Unpleasant odors					
Si	126 (65.60)	66 (34.40)	192	0.230 (0.62)	1.04 (0.88-1.22)
No	94 (63.10)	55 (36.90)	149		1
Visual contamination					
Yes	32 (65.30)	17 (34.70)	49	0.010 (0.90)	1.01 (0.81-1.26)
No	188 (64.40)	104 (35.60)	292		1
Air pollution					
Yes	199 (63.00)	117 (37.00)	316	4.470 (0.03)	0.74 (0.61-0.90)
No	21 (84.00)	4 (16.00)	25		1
Water pollution					
Yes	13 (81.30)	3 (18.80)	16	2.050 (0.15)	1.27 (0.99-1.63)
No	207 (63.70)	118 (36.30)	325		1
Others (smoke, noise, and vapors)					
Yes	9 (75.00)	3 (25.00)	12	0.590 (0.43)	1.16 (0.83-1.63)
No	211 (64.10)	118 (35.90)	329		1

95%CI = 95% confidence interval; PR = prevalence ratio. Values in bold: statistically significant association when p < 0.05.

### SOCIODEMOGRAPHIC, LABOR AND ENVIRONMENTAL CONDITIONS THAT EXPLAIN ESI AMONG WORKERS

One of the statistically significant variables (p < 0.05) to explain a higher prevalence of ESI is not having working license, showing that the prevalence of ESI was 43.0% lower [adjusted prevalence ratio (PR_adj_) = 0.57] among those who did not have such license, with a decrease of 24% [unadjusted prevalence ratio (PR_unadj_) = 0.81; 95%CI = 0.68-0.95; PR_adj_ = 0.57; 95%CI = 0.33-0.97) after adjustment for the remaining sociodemographic, labor and environmental conditions. Other variable that had a significant contribution to explain a lower prevalence of ESI was the fact of considering job tenure as fair, poor, and very poor, observing that there was a decrease in the prevalence of ESI after adjustment of this characteristic for the remaining variables, varying from 19.0% in unadjusted analysis to 43.0% after adjustment for the remaining study variables (PR_unadj_ = 0.81; 95%CI = 0.68-0.97; PR_adj_ = 0.57; 95%CI = 0.34-0.94) (Table 5).

**Table 5:** Sociodemographic, labor, and environmental conditions, and pollutant sources that contribute to explain eye and skin irritation in informal street vendors working in downtown Medellin and participating in the study, Medellin, Colombia, 2015-2019

Condition - characteristic	Unadjusted PR	95%CI		Adjusted PR	95%CI	
		LT	UT		LT	UT
Working license: no	0.81	0.68	0.95	0.57	0.33	0.97
Job tenure: fair, poor, very poor	0.81	0.68	0.97	0.57	0.34	0.94
Air pollution: yes	0.74	0.61	0.90	0.34	0.11	1.07
Only occupation: vendor	0.86	0.71	1.05	0.69	0.39	1.21
Daily working hours: between 4 and 8	0.86	0.69	1.06	0.86	0.47	1.55
Age (years) - comparison category: 18 to 29						
30 to 44	1.03	0.68	1.57	1.01	0.29	3.48
45 to 59	1.17	0.79	1.75	0.76	0.35	1.66
> 60	1.18	0.78	1.78	1.13	0.59	2.16
Water pollution: yes	1.27	0.99	1.63	2.75	0.67	11.19
Length of time working in the profession (years) - comparison category: 5 to 10						
11 to 20	0.97	0.75	1.27	1.41	0.51	3.91
21 to 30	1.22	0.95	1.57	0.89	0.43	1.83
> 30	1.07	0.81	1.41	1.77	0.83	3.78
Period of greater air pollution - noon and morning	1.07	0.94	1.23	0.97	0.6	1.56
Sex: male	1.13	0.97	1.32	1.4	0.86	2.29
Waste accumulation in EEVV containers: yes	0.48	0.25	0.95	0.85	0.17	4.26

95%CI = confidence interval; LT = lower threshold; PR = prevalence ratio; UT = upper threshold. Values in bold: statistically significant association when p < 0.05.

Air pollution and waste accumulation in containers provided by EEVV in the public space, which were found to be significantly associated with a lower prevalence of ESI in bivariate analyses, lost their statistical significance and exhibited changes in their ability to explain a lower prevalence of ESI. Conversely, air pollution strengthened their association with a lower prevalence of ESI (PR_unadj_ = 0.74; PR_adj_ = 0.34), a situation contrary to that observed with waste accumulation in containers provided by EEVV in the public space, which had no longer a significant association with a lower prevalence of ESI (PR_unadj_ = 0.48; PR_adj_ = 0.85) in adjusted analysis after adjustment for the remaining sociodemographic, labor and environmental conditions, as observed in Table 5.

Conversely, water pollution increased its explanatory ability in the multivariate model (PR_adj_ = 2.75) compared with bivariate analyzes (PR_unadj_ = 1.27), showing that the prevalence of ESI was 1.75 times higher among those exposed to this type of pollution (Table 5).

There was also a change in the results of the multivariate model with regard to length of time working in the profession, considering that unadjusted analyzed yielded a higher prevalence of ESI for workers with 21 to 30 years of profession (PR_unadj_ = 1.22), whereas adjusted analysis yield a higher prevalence of ESI for those with more 30 years of profession (PR_adj_ = 1.77), followed by those with 11 to 20 years (PR_adj_ = 1.41), and those with 21 to 30 years (PR_adj_ = 0.89). Thus, vendors working for more 30 years in the profession were shown to have a 77.0% higher prevalence of ESI compared with those working for shorter (5 to 10 years) in the profession after adjustment for the remaining variables included in the multivariate analysis (Table 5).

Finally, it was found that the prevalence of ESI for men increased by 27.0% after adjustment for the remaining variables included in the analysis (PR_unadj_=1.13; PR_adj_ = 1.40), with a prevalence of ESI 40% higher than that of women (Table 5).

## DISCUSSION

The prevalence of ESI in the study population was 65.4% (244), a result similar to that reported in a study in which occupational dermatitis was one of the most frequent work-related diseases, with a 60% likelihood of presenting with dermatitis in formal workers of developed countries^14^. It is also important to bear in mind that the most prevalent skin disease in the work context is contact dermatitis (“irritation”), with a prevalence ranging from 70 to 90%.^15,16^

However, evidence on these diseases in the workplace is still scarce for the formal sector of economy,^17^ and is even scarcer for informal workers, which turn street and sidewalks of the cities into their workplace. It was observed that most participants in the present study were men, had a mean age of 50 years, and did not have a partner. With regard to their labor conditions, the highest percentage of workers had been working in the profession from 11 to 20 years and worked more than 8 hours a day, results similar to those reported in the Colombian National Diagnosis of health and working conditions of people working in the informal sector of economy in 20 departments of Colombia.^18^ Conversely, Ballesteros et al, in 2005, describe that, in population of recyclers of Medellin, up to 93% of men and 82% of women worked from 4 to 12 hours a day.^19^

A study conducted in the department of Cauca in 2013 reported that the informal workers participating in the study were predominantly itinerant vendors (65.9%),^20^ unlike what was observed in the present working population, of which only 4.7% reported to be itinerant vendors. With regard to the type of products, nearly 60% sold goods and pots, a result similar to that found in other studies^18^.

With regard to environmental conditions and polluted areas or sites (pollutant sources) identified by the workers, it was observed that 50.2% of them perceived unpleasant odors, and 89.4% perceived that the air was polluted in the workplace and its surroundings. These findings are somewhat related to those reported by a study conducted in a market place in Bogotá, which investigated the conditions that informal workers perceived as dangerous for the development of their activities and observed that 47.3% reported unpleasant odors, 39.5% lack of order or cleanliness, and 30.4% the presence or rats^21^. The present study also observed that 86.6% of workers felt excessive hot or cold in their workplace. There are variable results in the literature, which reported a prevalence of 96.0% for workers involved in the production of fique in the municipality of Chachagui, located in department of Nariño.^22^

There was a significant association between ESI, job stability, and working license, with workers who considered to have fair, poor, or very poor stability showing a lower prevalence (19.0%) of ESI than those who considered to have good or very good stability. This finding is different from that reported in a study on the Indian informal sector,^23^ which found that job tenure is related with risk of developing diseases in informal workers, although not specifying the type of occupational diseases these workers developed. As for working license, it was found that those who did not have such license showed the lowest prevalence of ESI (19%). However, when comparing this finding with evidence reported in the literature, there was no studies reporting this type of data, a situation that leave open the possibility of further investigating these characteristics in future studies.

Conversely, waste accumulation in containers provided by EEVV was associated with a higher prevalence of ESI, a situation that, similar to the one mentioned above, has not been reported yet in the specialized literature. There was also an association between ESI and air pollution, in which, paradoxically, being in environments where vendors considered that air was polluted reduced the prevalence of eye and skin diseases by 26%, contrary to findings reported in the literature, which stated that passive smoking and polluted air was risk factors for developing eye diseases.^24^

Furthermore, a higher prevalence of ESI was observed among workers who reported to feel exaggerated heat or cold in the workplace, among those who reported to be exposed to chemicals, among those who perceived that their workplace was affected by other pollutants, such as smoke; and among vendors whose workplace was located near restaurants. These findings are similar to those reported in the literature, which showed that high temperatures, exposure to irritating chemical compounds^24^ and windy and dusty areas^25^ have been related to eye diseases.

With regard to personal protection mechanisms that are supposed to be essential factors in occupational health, they are not broadly used by the population of informal workers, which exacerbates their exposure to risk factors and predisposes to work accidents.^23^ The present study indicated that the use of personal protection measures reduces the risk of developing cutaneous and ocular manifestations by 2.0%. Similar results were reported by Ballesteros et al. in the city Medellin in 2005 with a population of recyclers in downtown Medellin, of which less than 52.0% used the required protection measures, thus increasing the likelihood of developing respiratory and gastrointestinal diseases, among others.^19^ A meta-analysis on informal work found that waste collectors do not often use any type of personal protection equipment, and they frequently use their hands to collect the recyclable material; one of the studies revealed that half of the collectors (56%) reported never having worn gloves during work.^4^ Conversely, with regard to labor conditions, a clean, tidy workplace favors a lower prevalence of eye diseases.^25^. Similar data were observed in the present study, in which an 11.0% lower prevalence of conjunctivitis was observed among worker who reported to have a clean, tidy workplace.

The facts of considering job tenure as fair, poor, or very poor and not having a working license explained a lower prevalence of eye and skin irritation. However, no reports have been identified in the literature that allow to assert which factors explain a lower or a higher prevalence of this disease for informal workers. The following variables were also able to explain a higher prevalence of ESI: contact with polluted water, length of time in the professional greater than 30 years, male sex, street vending as only profession, working from 4 to 8 hours a day, and observing waste in containers of the Medellin public service company named EEVV. These are some of the characteristics reported for the general population, as shown by a study of pediatric populations in India and in low and medium-income countries, which observed that the impact of water, sanitation and hygiene interventions reduced the risk of conjunctivitis by 51% (risk reduction = 0.49; 95%CI = 0.45-0.55).^26^ Consistent with this, the present study showing that being near polluted water increases the prevalence of ESI by 175% (PR_adj_ = 2.75). This study also found that working for more than 30 years in the profession is able to explain a greater IOP, a result comparable with that reported in an article on environmental health and preventive medicine which observed that working for more than10 years in the profession was a risk factor for occupational dermatitis ocupacional.^15^ Additionally, another paper found that there is a high prevalence of tear secretion dysfunction (risk factor for conjunctivitis) among female workers who perform lights tests; moreover, this prevalence increases as length of time in the professional increases.^24^

Male sex was able to explain higher prevalence of ESI, in agreement with results observed in the literature showing that male sex was associated with a higher prevalence of occupational dermatitis, since men accounted for 64.0% of individuals with this disease.^16^ The same prevalence was also found in a study assessing the clinical and sociodemographic profile of patients with occupational dermatitis in a dermatology service.^27^ However, other studies with formal workers reported different findings, with a 70.0% higher prevalence of dry eye among women.^24^

With regard to age, a study reported that there is a higher prevalence of dry eye (up to 4.34%) among men older than 50 years,^24^ differently from the results of some studies which found a higher prevalence of occupational dermatitis among individual younger than 50 years of age.^28,29^

### LIMITATIONS

There were difficulties in comparing some environmental and labor characteristics and conditions associated with ESI, given the scarcity of scientific evidence related to informal workers or to other types of jobs, and although the type of design only allows us to speak of factors associated and that explain prevalences these results. Moreover, study design limited statistical analyses. Both situations strengthen lines of research with this working population.

## CONCLUSION

It was found that the population of informal street vendors in downtown Medellin are directly exposed to certain environmental factors and labor conditions that are able to shape its condition of socio-labor and environmental vulnerability. This study highlights the importance, in terms of public health, of conducting relevant interventions in occupational health that have a positive impact on workers’ life and health.
